# People with intellectual disabilities living in care facilities engaging in virtual social contact: A systematic review of the feasibility and effects on well‐being

**DOI:** 10.1111/jar.12926

**Published:** 2021-07-09

**Authors:** Lianne Bakkum, Carlo Schuengel, Paula S. Sterkenburg, Noud Frielink, Petri J. C. M. Embregts, Johanna Clasien de Schipper, Annet ten Brug, Anne Tharner

**Affiliations:** ^1^ Department of Clinical Child and Family Studies and Amsterdam Public Health Research Institute Vrije Universiteit Amsterdam Amsterdam The Netherlands; ^2^ Academische Werkplaats 's Heeren Loo – VU Vrije Universiteit Amsterdam Amsterdam The Netherlands; ^3^ Department of Public Health and Primary Care University of Cambridge Cambridge UK; ^4^ Bartiméus Zeist The Netherlands; ^5^ Academische Werkplaats Sociale relaties en gehechtheid, Bartiméus – VU Vrije Universiteit Amsterdam Amsterdam The Netherlands; ^6^ Academische Werkplaats Leven met een verstandelijke beperking, Tranzo, Tilburg School of Social and Behavioral Sciences Tilburg University Tilburg The Netherlands; ^7^ Academische Werkplaats EMB University of Groningen Groningen The Netherlands

**Keywords:** COVID‐19, information and communication technology, interpersonal relationships, social contact, systematic review, well‐being

## Abstract

**Background:**

During the initial phase of the COVID‐19 pandemic, many people with disabilities living in home care facilities could not receive visitors. The use of virtual social contact has been recommended by health authorities. This systematic review examined the scientific evidence of the use and feasibility of information and communication technology (ICT) for social contact by people with intellectual disabilities living in care facilities, and potential effects on well‐being.

**Methods:**

Five databases were searched using traditional systematic screening and machine‐learning supported screening. Findings are presented in a narrative synthesis using thematic analysis.

**Results:**

Nine studies were included. We described three themes: means of ICT used for social contact; effects on well‐being; and benefits, barriers, and preconditions.

**Conclusions:**

Engaging in virtual social contact may be feasible for people with severe to mild intellectual disabilities, but there is little concrete evidence that this can be used as an alternative for in‐person contact.

## 1 INTRODUCTION

To limit the spread of the SARS‐CoV‐2 virus and COVID‐19, national governments took drastic measures over the course of 2020, such as restriction of travel, closure of schools, and the advice to quarantine in case of exposure or infection. Besides measures for the general population, actions were taken internationally to protect vulnerable people from getting infected. Among other measures, restrictions were placed on visits to long‐term care facilities, such as those for people with disabilities (Salcher‐Konrad et al., 2020; World Health Organization, 2020). Many people with disabilities living in home care facilities could therefore not receive visitors or visit anyone from outside their facility. But especially during this time of anxiety and disrupted routines, residents' need for contact with loved ones would likely have been heightened (Embregts et al., 2020; Schuengel et al., 2013). Adults with mild intellectual disability indicated that they missed having in‐person contact with family and friends during the first COVID‐19 lockdown period in the Netherlands (Embregts et al., 2020). In addition, family and friends often play a significant role in the emotional and practical support of people with intellectual disabilities (Giesbers et al., 2019; Van Asselt‐Goverts et al., 2013), also during the pandemic (Redquest et al., 2020). Given the potential impact of the pandemic measures on people with intellectual disabilities living in care facilities (Schuengel et al., 2020), it may be important for their well‐being to maintain contact with friends and family.

The use of virtual social contact, such as telephoning and video conferencing, has been widely recommended as an alternative way to stay in contact during the pandemic (e.g., World Health Organization, 2020). Previous studies have discussed the use of information and communication technology (ICT) by people with intellectual disabilities (e.g., Chadwick et al., 2013), but research focusing on ICT for social contact is limited (see Barlott et al., 2020). People with intellectual disabilities increasingly use the internet, but may experience more barriers with using it than people without intellectual disabilities (Chadwick et al., 2013). On the other hand, the use of ICT may positively impact the well‐being of people with intellectual disabilities (e.g., Caton & Chapman, 2016; Den Brok & Sterkenburg, 2015; Dyzel et al., 2020; Oudshoorn et al., 2020). The aim of this systematic review was to gain insight into the available scientific evidence on the use and feasibility of virtual social contact by people with intellectual disabilities living in home care facilities, and the potential to mitigate the impact of restricted in‐person visiting policies. The following questions oriented our selection and extraction of the literature:

- Which ICT‐mediated means for social contact are available to people with disabilities living in home care facilities or supported living arrangements, and are being used by people with disabilities for social contact?
- What are the effects of using ICT for social contact on wellbeing, quality of life, and quality of interpersonal relationships in the context of restricted in‐person visiting policies?
- How do people with disabilities, family and other social network members and professionals perceive the benefits, barriers, and preconditions of using ICT for social contact?
- Are use, effects, and perceived benefits, barriers and preconditions different with age, type of disability, level of disability, off‐line level of engagement, and reasons for restricted in‐person visiting policies?

## 2 METHOD

This review followed from a research question developed together with the Dutch Association of Healthcare Provides for People with Disabilities (Dutch abbreviation: VGN) and the client advocacy body KansPlus. PROSPERO registration: https://www.crd.york.ac.uk/prospero/display_record.php?ID=CRD42020186442.

### 2.1 Eligibility criteria

Inclusion criteria were specified using the PICO format (i.e., population, intervention, comparison, outcome; Liberati et al., 2009): (a) the population included children, adolescents, and adults with intellectual disabilities (borderline, mild, moderate, severe, profound), motor disability, autism, visual impairment, auditory impairment, deaf‐blindness, acquired brain injury, and multiple disability, living in care facilities; broad population criteria were used because we expected few relevant studies and because the findings were expected to be relevant for all people with (intellectual) disabilities living in care facilities; (b) interventions included any form of ICT‐mediated social contact between a person with disability living in a care facility with another person who lives outside the care facility, occurring through different modalities, such as video, audio, photo sharing, and text messaging; (c) ICT‐mediated contact was used as an alternative for in‐person contact, while in‐person contact was restricted by (emergency) policy of the government or care facility; (d) outcomes included well‐being, quality of interpersonal relationships, quality of life, and perceived benefits, barriers, and preconditions of using ICT. During the screening, we dropped part of the criterion under (c) that ‘in‐person contact is restricted by (emergency) policy of the government or care facility’, because it applied to none of the otherwise eligible studies.

Studies were included if targeted at people living in home care facilities. We used a broad operationalisation of care facilities. This included home care facilities with 24‐h support and people living in small‐group supported living arrangements. We also included studies in which part of the sample lived in a care facility.

The following exclusion criteria applied: children, adolescents, and adults with disabilities living with their parents or other family members; people with disabilities only attending day care facilities; and elderly with neurodegenerative diseases.

### 2.2 Search strategy

We searched MEDLINE, Web of Science, Scopus, LENS.org, and IEEE Xplore for original studies published until May 2020, in English, Dutch, or German. Both published and unpublished studies were sought, and no limitations were imposed on the study designs eligible for inclusion. The last search took place on 15 January 2021. The search protocol can be found on Open Science Framework: https://osf.io/byxh8/ and as a Data S1.

### 2.3 Study selection

The study selection was conducted using a novel two‐phase approach: a first, manual selection of studies and a second, broader selection of studies using active systematic review software (ASReview; ASReview Core Development Team, 2019). ASReview uses machine learning combined with active learning to predict which studies are most likely to be included, based on prior decisions by the author of whether presented studies were considered relevant or irrelevant. This made it feasible to screen a large set of additional literature, decreasing the risk of false negatives. The search strategy in the second selection was broadened by dropping the C (comparator) terms because these related to residential care and restrictive policies, which were not essential for addressing the research questions. This yielded a larger selection of additional candidate studies.

**FIGURE 1:**
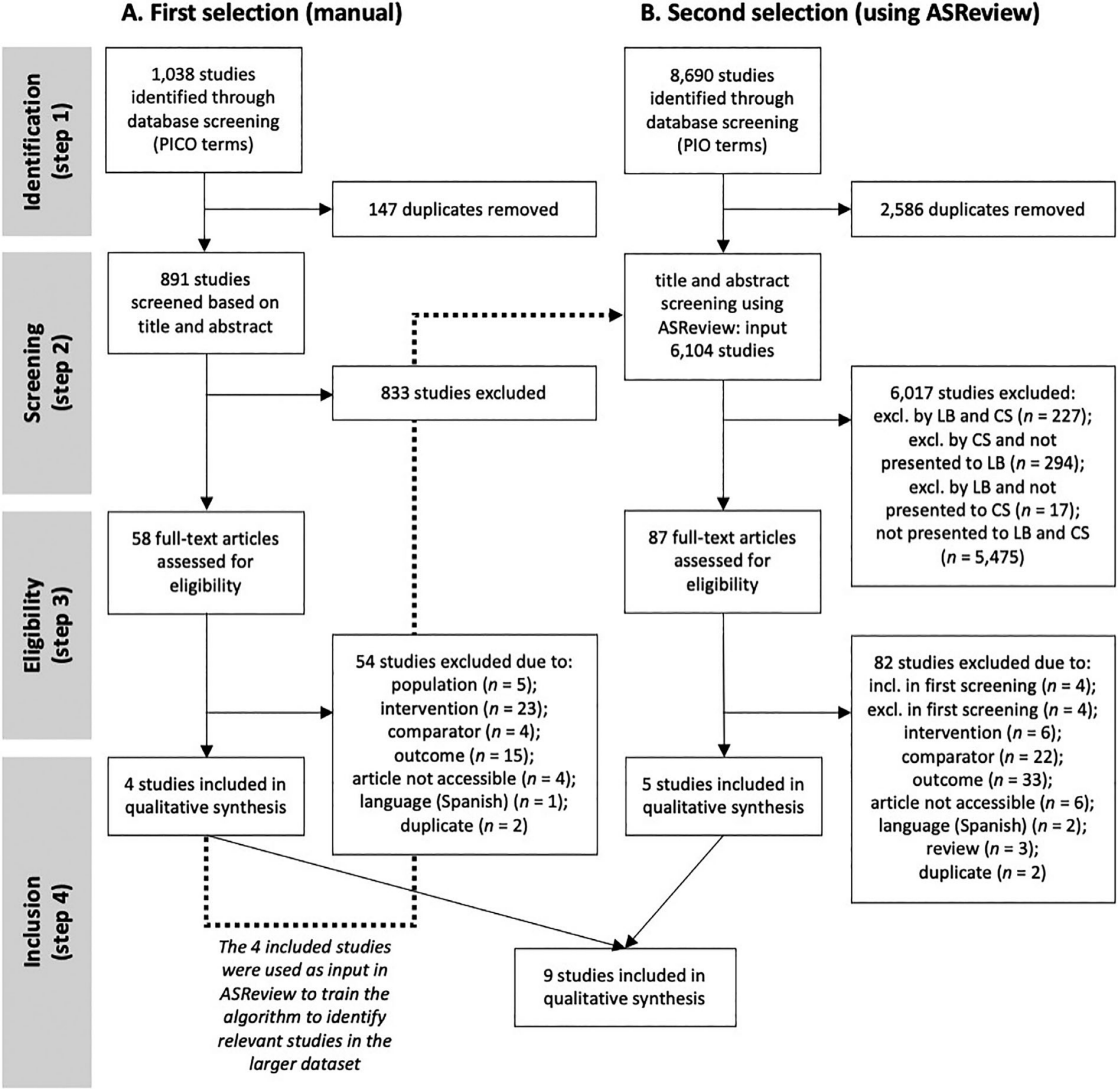
PRISMA flow diagram. The left side of the figure (a) describes the first, manual selection of studies. The right side (b) describes the second, broader selection of studies (using ASReview software)

The first selection was conducted as follows (Figure 1a): (a) databases were searched using the PICO terms, references were imported into *Zotero* referencing software and duplicates were removed; (b) all titles and abstracts were manually screened independently by two reviewers using the Rayyan application (Ouzzani et al., 2016); (c) full‐texts were assessed for eligibility by one reviewer; (d) a preliminary selection was made through discussion by two reviewers. For the second selection of studies, the following steps were used (Figure 1a): (a) databases were searched using the PIO terms (C terms dropped); (b) titles and abstracts were screened independently by two reviewers using ASReview (screening time: 3 h); (c) full‐texts were assessed for eligibility by one reviewer; (d) a preliminary selection was made through discussion by two reviewers. The final selection was made by these two reviewers, with consensus reached through discussion.

### 2.4 Data extraction

Two reviewers independently extracted the data using Google Forms, with discrepancies solved through discussion. We extracted general information about the studies, that is, bibliographic information, design, method (quantitative, qualitative, mixed/multiple methods), and method(s) of assessment. To answer the research questions, we extracted information about the target population, sample characteristics, reasons for restricted in‐person contact, means of ICT used for social contact, support from the care facility with using ICT, independent and dependent variables (quantitative studies), definition of outcomes (qualitative studies), informants, and information about the nature/goal of the social contact, impact of restricted contact on well‐being, the level of ‘off‐line’ engagement between interactive partners, and perceived benefits, barriers and preconditions of using ICT for social contact by people with disabilities and their social network members. We also recorded any additional outcomes that were measured, such as the use of ICT for other purposes than communication. Criteria were additionally reviewed for necessity and completeness by another reviewer before extraction.

### 2.5 Data synthesis

The findings are presented in a narrative synthesis, based on a deductive thematic analysis following the steps described by Braun and Clarke (2006). The themes were defined prior to analysis, based on the review questions. The initial coding and analysis was done by two reviewers, who discussed the content of the themes and subthemes in two meetings, reaching consensus after the second meeting. Because only four out of nine studies presented quantitative findings, on different outcomes, insufficient data were available for a quantitative synthesis.

## 3 RESULTS

During the first selection (Figure 1a; PICO terms), two reviewers independently reviewed the titles and abstracts of all 891 potentially eligible studies. Using the Rayyan application, articles were rated as ‘included’, ‘maybe’, or ‘excluded’. For the inter‐rater reliability, articles rated as maybe were re‐coded as included, yielding an agreement of 55% (*κ* = 0.547). One reviewer reviewed the full‐texts of the agreements and mismatches (*k* = 58), after which a selection of four eligible studies was made through discussion with a second reviewer. During the additional, broader selection (Figure 1b; PIO terms), two independent reviewers screened *k* = 328 (first reviewer) and *k* = 610 studies (second reviewer) using ASReview (rated as ‘relevant’ or ‘irrelevant’). One reviewer reviewed the full texts of the agreements and mismatches (*k* = 87), and a selection of five additional studies was made after discussion with a second reviewer. In total, nine studies were eligible for inclusion.

The characteristics of the included studies are presented in Table 1. Studies were published between 2006 and 2019. Methods were qualitative or quantitative descriptive. Three studies were described as single‐case experiments (Lancioni et al., 2015; Lancioni, Singh, O'Reilly, Sigafoos, Oliva, & Campodonico, 2013; Lancioni, Singh, O'Reilly, Sigafoos, Oliva, Campodonico, D'Amico, et al., 2013), but were considered quantitative descriptive because results for the different conditions were described without statistical tests. One study (Shpigelman & Gill, 2014) described their method as a mixed method approach, but as the qualitative and the quantitative parts addressed different questions instead of using multiple methods to answer the same questions and aggregating the results, the study was considered as partly quantitative descriptive and partly qualitative.

**TABLE 1:** Characteristics of included studies

Authors	Design	Methods	Target population	Sample size	Age of target population (years)	ICT used for social contact	Main findings	Quality appraisal (MMAT)
Lancioni, Singh, O'Reilly, Sigafoos, Oliva, Campodonico, D'Amico, et al. (2013)	Single‐case experiment, descriptive	Observations, computer‐recorded responses	Adults with severe multiple disabilities	2	23 and 36	Telephone (touchpad or computer mouse during baseline, and camera‐based or wobble microswitches during the intervention sessions)	The participants made more frequent and longer phone calls during the intervention sessions, as compared to baseline. They also showed preference among telephone partners and had larger percentages of observation intervals with smiles during the intervention sessions. Participants did not make any phone calls during the baseline sessions.	20%
Lancioni, Singh, O'Reilly, Sigafoos, Oliva, and Campodonico (2013)	Single‐case experiment, descriptive	Observations, computer‐recorded responses	One child and one adult with severe multiple disabilities	2	11 and 21	Telephone (standard telephone device during baseline, and optic microswitches during the intervention sessions)	The participants made more frequent and longer phone calls during the intervention sessions, as compared to baseline. They also showed preference among telephone partners and had larger percentages of observation intervals with smiles during the intervention sessions. Participants did not make any phone calls during the baseline sessions.	20%
Lancioni et al. (2015)	Single‐case experiment, descriptive	Computer‐recorded responses	Adults with severe multiple disabilities	4	31, 39, 63, and 81	Video (pre‐recorded videos with distorted sounds or music/sports/events/family members talking, during the baseline and intervention sessions)	The participants produced more responses (by activating the microswitch) after preferred stimuli (e.g., videos of family members talking) as compared to the non‐preferred stimuli (videos with distorted or blurred sounds), during the intervention sessions.	20%
Parsons et al. (2006)	Qualitative	Observations, interviews with target population and staff	Adults with intellectual disabilities	No information	No information	Digital photos, e‐mail, internet (pre‐social media era), writing software	The most frequently reported types of ICT used for communication by participants living in care facilities were main stream software programmes, such as email and the internet.	20%
Patterson and Potter (2009)	Case study, qualitative	Analysis of sections of pre‐closing sections of telephone calls	Young adult with moderate intellectual disability and autism	1	19	Telephone	The study described closing sections of telephone calls that may build and protect relationships between a person with intellectual disability living in a care facility and her family members.	100%
Ramsten et al. (2019)	Qualitative	Focus groups and individual interviews with professional carers	Young adults with mild‐to‐moderate intellectual disabilities	17	18–30	Social media, videoconferencing, telephone, digital photos, text messaging	One main theme was identified: enforcing a balance between social inclusion and social exposure. Three subthemes were identified: enabling participation in social life, enabling independence and practical actions, and bringing out the vulnerability in a risky environment.	80%
Ramsten et al. (2020)	Qualitative	Interviews with target population	Young adults with mild‐to‐moderate intellectual disabilities	11	22–31	Social media, telephone, text messaging	The participants used ICT for leisure time activities, which was the main theme of the findings. Two generic themes split into two subthemes were identified: Social relationships (subthemes: Family relationships and daily support, interactions based on interests), and solitary pastime (amusement, support for offline activities).	100%
Shpigelman (2018)	Qualitative	Observations and interviews with target population	Adults with mild‐to‐moderate intellectual disabilities	20	58% were 30 years or older	Facebook	Five themes were identified: an opportunity to be like (non‐disabled) others, becoming a member of the community, becoming visible to others, increasing one's popularity, and positive vs. negative feelings.	100%
Shpigelman and Gill (2014)	Quantitative descriptive and qualitative	Survey completed by target population	Adults with intellectual disabilities (estimated mild‐to‐moderate)	58	21–43	Facebook	The quantitative results indicated that participants had positive and negative perceptions of and experiences with Facebook. In the qualitative part of the study, participants suggested ways of making Facebook more accessible for users with intellectual disabilities.	80% qualitative criteria, 20% quantitative descriptive criteria

*Note*: MMAT, mixed methods appraisal tool (Hong et al., 2018).

Two independent reviewers assessed the quality of the included studies using the mixed methods appraisal tool (MMAT; Hong et al., 2018; see Table 1). The screening criteria of the MMAT were met by all studies, except for the study by Lancioni et al. (2015). In this study, the research questions were not clearly formulated. We still decided to include it, because it is an empirical study and the findings were relevant to our review questions. Three studies (Patterson & Potter, 2009; Ramsten et al., 2020; Shpigelman, 2018) met all design‐specific criteria. One study (Ramsten et al., 2019) met 80% of criteria, but showed discrepancies between the data analysis and interpretation of results. Another study (Shpigelman & Gill, 2014) met 80% of the criteria for qualitative studies and 20% of the criteria for quantitative descriptive studies. The remaining studies had several methodological problems, such as risk of bias in the data collection methods and a lack of information about the derivation and interpretation of results (Parsons et al., 2006), and risk of nonresponse bias, lack of information regarding the sampling strategy, representativeness of the samples, outcome variables, and inappropriate statistical tests (Lancioni et al., 2015; Lancioni, Singh, O'Reilly, Sigafoos, Oliva, & Campodonico, 2013; Lancioni, Singh, O'Reilly, Sigafoos, Oliva, Campodonico, D'Amico, et al., 2013). The complete quality assessment of the included studies is provided in the Appendix.

All studies addressed adults (>18 years) with intellectual or multiple disabilities. One study included adults and a child with multiple disabilities including intellectual disability (Lancioni, Singh, O'Reilly, Sigafoos, Oliva, & Campodonico, 2013). Three studies included people with severe multiple disabilities (Lancioni et al., 2015; Lancioni, Singh, O'Reilly, Sigafoos, Oliva, & Campodonico, 2013; Lancioni, Singh, O'Reilly, Sigafoos, Oliva, Campodonico, D'Amico, et al., 2013) and four studies included people with mild to moderate intellectual disabilities (Patterson & Potter, 2009; Ramsten et al., 2019, 2020; Shpigelman, 2018). In one study (Shpigelman & Gill, 2014), the level of intellectual disability of the target population was not specified. However, given that the study was targeted at adults who were ‘capable of using the internet’, we estimated the target population to be people with mild‐to‐moderate intellectual disabilities. One study (Parsons et al., 2006) provided no further information about the sample. Studies were conducted by researchers from Israel, Italy, New Zealand, Sweden, the Netherlands, the United Kingdom, and the United States.

### 3.1 Main findings

Three themes with subthemes were described: (a) means of ICT used for social contact (subthemes: direct reciprocal means of communication, indirect means of communication, differences in use regarding type and level of disability); (b) effects on well‐being (subthemes: emotional well‐being and quality of life, quality of interpersonal relationships); and (c) benefits, barriers, and preconditions (subthemes: benefits, barriers, preconditions; Table 2).

**TABLE 2:** Identified themes and subthemes across the included studies

Theme	Subtheme
1. Means of ICT used for social contact	1.1 Direct reciprocal means of communication
	1.2 Indirect means of communication
	1.3 Differences in use regarding type and level of disability
2. Effects on well‐being	2.1 Emotional well‐being and quality of life
	2.2 Quality of interpersonal relationships
3. Benefits, barriers, and preconditions	3.1 Benefits
	3.2 Barriers
	3.3 Preconditions

Abbreviation: ICT, information and communication technology.

### 3.2 Theme 1: Means of ICT used for social contact

This theme describes the means of ICT used for social contact with family, friends, and other significant persons outside of the care facility.

#### 3.2.1 Subtheme 1.1: Direct reciprocal means of communication

##### Telephone

Young adults with mild‐to‐moderate intellectual disabilities in the study by Ramsten et al. (2020) used the telephone to contact support staff and to keep family members informed about their well‐being. Patterson and Potter (2009) analysed the closing sections of 52 telephone calls (i.e., the parts where the first mention of closing the call was made until the actual closing of the call) between a young adult with intellectual disability and autism and her family members, and described several closings of phone calls that may protect and improve the quality of interpersonal relationships. Use of the telephone by people with severe multiple disabilities has also been described. In the studies by Lancioni, Singh, O'Reilly, Sigafoos, Oliva, Campodonico, D'Amico, et al. (2013); Lancioni, Singh, O'Reilly, Sigafoos, Oliva, and Campodonico (2013), people with severe multiple disabilities were provided with telephone microswitch technology helping them to choose which family member or friend to call. Microswitches were attached to the participants' mouth or face or placed nearby the hand, and were activated by small facial, head, mouth opening, or hand movements. A computer presented the names of persons to call, after which participants could respond by activating the microswitch, indicating if they wished to call the person.

##### Video

In the study by Lancioni et al. (2015), four adults with severe multiple disabilities used microswitch technology to choose between videos with distorted sounds (non‐preferred videos) or videos with music/sports/events/family members talking (preferred videos). If participants responded within 6 s of the video by activating the microswitch, the computer continued to present the video for 20 s. If participants did not respond, the computer paused and presented the next video. In the intervention sessions, participants more often activated the microswitch after preferred videos (including videos of family members) than non‐preferred videos, suggesting purposeful choice behaviour. Also, support staff perceived that the use of video conferencing by young adults with mild‐to‐moderate intellectual disabilities was a way to have frequent contact with friends and family, and that this decreased loneliness (Ramsten et al., 2019).

#### 3.2.2 Subtheme 1.2: Indirect means of communication

Text messaging, social media, email, and other (pre‐social media) software were identified as more indirect and distant ways of communication than telephoning and video conferencing. Also, these means of communication were identified as less reciprocal than telephoning and videoconferencing. For example, people can respond to messages on social media without receiving a reply, or receive a text message without responding.

##### Text messaging

Young adults with mild‐to‐moderate intellectual disabilities in the study by Ramsten et al. (2020) received text messages from family members and staff, which was perceived as a ‘positive way of receiving information’. (p. 8). Some participants reported problems with understanding how to text message and responded by voice calling instead. Support staff perceived that the use of text messaging to contact family and friends decreased loneliness in this population (Ramsten et al., 2019).

##### Social media

In the study by Ramsten et al. (2020), the majority of young adults with intellectual disabilities owned a smartphone and a computer. They followed friends and family on Facebook and Instagram and connected with them by chatting and liking and responding to photos and timeline messages. Facebook was also used to initiate new relationships with unknown people, based on similar interests. More than half of adults with intellectual disabilities in the survey study by Shpigelman and Gill (2014) visited Facebook at least once per week, and mostly used it for contacting people they also met offline, such as friends and family. These uses of Facebook were also described by Shpigelman (2018), who conducted observations and interviews with adult Facebook users with intellectual disabilities.

##### Email and other software (pre‐social media)

Parsons et al. (2006) conducted their study before social media were widely adopted. In this context, the internet and email were among the most frequently used types of ICT used for contact with family and friends. In addition, word processing software was used to write letters to loved ones, and the digital camera was used to share photos.

#### 3.2.3 Subtheme 1.3: Differences in use regarding type and level of disability

**FIGURE 2:**
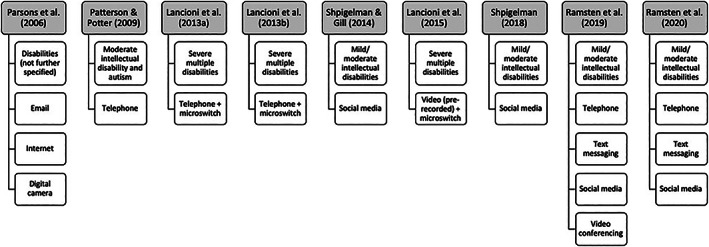
Included studies, target populations, and means of information and communication technology used for social contact. Studies are presented in ascending order of publication year

This subtheme describes differences in use with regard to type and level of disability. Initially, our focus was on a variety of factors that may affect the use, effects, and feasibility of the use of ICT by people with disabilities, including but not limited to age, type and level of intellectual disability, off‐line level of engagement, presence of restricted in‐person visiting policies, and reasons for these measures. Based on the included studies, not all of these factors could be examined. However, differences in use regarding type and level of disability were described in the included studies. Figure 2 provides an overview of the studies, target populations, and means of ICT used for social contact. Overall, people with mild‐to‐moderate intellectual disabilities were able to use relatively advanced technology such as social media and other consumer technology, even though support may be needed to overcome practical difficulties, such as help with setting up accounts and learning how to use devices. People with severe multiple disabilities were able to independently make telephone calls and choose to watch videos of family members, but only after extensive training using microswitch technology.

### 3.3 Theme 2: Effects on well‐being

The initial focus on well‐being in the context of restricted visiting policies had to be abandoned because it was not studied. Instead, we focused on the effects of using ICT for social contact on the well‐being of users.

#### 3.3.1 Subtheme 2.1: Emotional well‐being and quality of life

##### Positive effects

Lancioni et al. observed people with severe multiple disabilities in baseline conditions in which they used a touchpad or a mouse to make a call (Lancioni, Singh, O'Reilly, Sigafoos, Oliva, Campodonico, D'Amico, et al., 2013) or were provided with a standard telephone device and helped by a research assistant to place a call ‘to limit frustration’ (Lancioni, Singh, O'Reilly, Sigafoos, Oliva, & Campodonico, 2013), and in experimental conditions in which participants used microswitches to make telephone calls independently. In both studies, participants increased their smiling, interpreted as ‘indices of happiness’ (Lancioni, Singh, O'Reilly, Sigafoos, Oliva, & Campodonico, 2013, p. 4181), during the intervention sessions compared to the baseline condition. However, participants made no phone calls during the baseline sessions and it was unclear whether they had the ability to use the standard telephone device. Family and support staff indicated feeling positive about the intervention and mentioned it helped them to feel more involved (p. 4182). One participant in the study by Lancioni et al. (2015) activated her microswitch with a smile, and showed more smiles after preferred videos (including videos of family members) as compared to videos with distorted sounds.

Support staff perceived that independent use of ICT for social contact may decrease loneliness in young adults with mild‐to‐moderate intellectual disability (Ramsten et al., 2019). Adults with intellectual disabilities in the study by Shpigelman and Gill (2014) reported both positive and negative feelings and experiences with using Facebook. The majority (82%) reported feeling more comfortable talking to people on Facebook, instead of face‐to‐face, and responses to the open‐ended questions indicated positive effects on their emotional well‐being. According to Shpigelman (2018), the use of Facebook contributed to the ‘general psychological well‐being’ of adults with intellectual disabilities. The participants reported feelings of ‘mood elevation, happiness, laughter, and pleasure’ after using Facebook (p. e84). The use of Facebook was also described as having positive effects on participants' sense of being part of the community.

##### Negative effects

Adults with intellectual disabilities responded that Facebook had negative effects on their well‐being (e.g., 74% of participants reported not feeling like everyone else when using Facebook, and 88% did not enjoy using Facebook; Shpigelman & Gill, 2014). Respondents in another study (Shpigelman, 2018) reported feelings of stress and frustration when experiencing ‘technical and conceptual difficulties’ (p. e84) while using Facebook. Young adults with intellectual disabilities perceived it as ‘annoying’ when the use of social media and text messaging was hindered by technical problems, such as an unresponsive touch screen (Ramsten et al., 2020).

#### 3.3.2 Subtheme 2.2: Quality of interpersonal relationships

The case study by Patterson and Potter (2009) described examples of closing sections of telephone calls between a young adult with an intellectual disability and autism living in a care facility and her family members. According to the authors, the way in which a telephone call is ended may protect and improve the quality of the relationship between a person with intellectual disability and a person without intellectual disability. An example of a telephone closing that protects the quality of interpersonal relationships is as follows: the speaker announces that they intend to close the call, but that this is not something that they desire. They might tell their loved one to leave the call because they need to do something else. This shows reluctance to close the call, which indicates responsiveness and care for the other person (Patterson & Potter, 2009). This case study not only indicates that the telephone can be used to maintain contact with distant family members, but also that the quality of the call might be important for building interpersonal relationships. Ramsten et al. (2020) reported that young adults' use of ICT and social media to contact friends living in other cities ‘increased the opportunities for social interactions and helped to maintain and deepen relationships with friends’ (p. 8). People with severe multiple disabilities (Lancioni, Singh, O'Reilly, Sigafoos, Oliva, & Campodonico, 2013; Lancioni, Singh, O'Reilly, Sigafoos, Oliva, Campodonico, D'Amico, et al., 2013) showed preferences for certain telephone partners, some were called more frequently than others.

### 3.4 Theme 3: Benefits, barriers, and preconditions

This theme with subthemes describes how people with intellectual disabilities, family, and professionals perceived the benefits, barriers, and preconditions of using ICT for social contact.

#### 3.4.1 Subtheme 3.1: Benefits

##### Consumer technology

The use of consumer technology for social contact has been reported by most of the included studies. Social communication technology has seen a large number of innovations over the past roughly 15 years. Before services such as social media were widely adopted, Parsons et al. (2006) pointed out that mainstream programmes or devices were the most frequently reported uses of ICT by people with disabilities attending day care facilities or living in residential care. The authors suggested that this may reflect organisational decisions to provide users with certain computer packages and that ‘mainstream programmes may be sufficient for the provision of varied and interesting ICT use and may be achieved without necessarily having to find additional funds for learning disability specific software’, but also recognised that specialised software ‘may enhance ICT provision by catering to more specific needs’. (p. 40). However, no sample characteristics were reported in this study. Fourteen years after the study by Parsons et al. (2006), Ramsten et al. (2020) concluded that the use of text messaging and social media by young adults with mild‐to‐moderate intellectual disability seemed ‘mainstream’, but that it was more limited than in the general population, ‘because cognitive impairment increases the need for support’ (p. 15). In this population, ICT was also used for other activities than social contact, such as playing games and listening to music (Ramsten et al., 2019).

#### 3.4.2 Subtheme 3.2: Barriers

##### Practical difficulties

All adults with intellectual disabilities in the study by Shpigelman (2018) used Facebook independently, but also reported barriers. These included practical issues, such as problems with creating an account and understanding Facebook jargon, and literacy difficulties, such as using functions that require typing and reading long posts of other users. Similarly, some young adults in the study by Ramsten et al. (2020) reported problems with understanding how to text message, but seemed to solve this by responding to text messages with voice calling. Difficulties with spelling did not seem to hinder their use of text messaging. Other barriers related to the use of a mobile phone included problems with the device or service provider. Although participants were often able to solve ‘simple technical problems’ with their phone (e.g., replacing the battery), technical issues sometimes led to problems with initiating contact with friends.

##### Perceived risks

Support staff perceived the concern that ICT, especially social media, is a risky environment for young adults with mild‐to‐moderate intellectual disability. These individuals were believed to be more socially vulnerable than the general population due to difficulties with social skills and social responsibility. Potential risks perceived by support staff included harassment, victimisation by fraud, and circulation of personal information (Ramsten et al., 2019).

#### 3.4.3 Subtheme 3.3: Preconditions

##### Implementation

Studies by Ramsten et al. (2019, 2020) described that most young adults with mild‐to‐moderate intellectual disabilities used their own devices such as smartphones and computers. Other technology may be less widely available, such as microswitches for independent use of ICT by people with severe multiple disabilities, as described by Lancioni, Singh, O'Reilly, Sigafoos, Oliva, Campodonico, D'Amico, et al. (2013); Lancioni, Singh, O'Reilly, Sigafoos, Oliva, and Campodonico (2013). However, according to the authors, such technology is easily implemented and can be used throughout the day, depending on the availability of family members and friends, and ‘with minimal time investment from caregivers or staff in general’ (Lancioni, Singh, O'Reilly, Sigafoos, Oliva, Campodonico, D'Amico, et al., 2013, p. 3195). The costs of these microswitch devices were estimated at around 2.000 US dollars per device.

##### Support from organisations, support staff, and family

Support from staff and family members were preconditions for the use of ICT by people with intellectual disabilities. Lancioni, Singh, O'Reilly, Sigafoos, Oliva, Campodonico, D'Amico, et al. (2013); Lancioni, Singh, O'Reilly, Sigafoos, Oliva, and Campodonico (2013) suggested that for people with severe multiple disabilities, social contact through technology is possible and appears pleasurable if they are provided with a person‐specific adapted device as well as intensive training with this device. With help from research assistants, people with severe multiple disabilities learned how to use the microswitch telephone technology and were able to successfully use it during the intervention sessions and even after the interventions. However, data from the post‐intervention sessions were not reported. Ramsten et al. (2019) noted that social care organisations for people with disabilities in Sweden did not have comprehensive plans for ICT support. Therefore, support for the use of ICT might depend on the interests of individual support staff members. However, support from family and staff with the use of ICT by young adults with mild‐to‐moderate intellectual disability seemed to be lacking in these studies. Ramsten et al. (2020) reported that some young adults did not know who to ask for help when experiencing difficulties with the use of social media. According to the authors, this may indicate that support was not offered by either family or support staff. In addition, participants sometimes received an evasive answer when asking for help. Support staff mentioned having ‘only superficial insight into the young adults' ICT use’ (Ramsten et al., 2019, p. 173), because they believed that the young adults preferred to ask friends and family for ICT support, instead of asking staff for help. In addition, the young adults' use of ICT was perceived as being dependent on the interests and attitudes of (older) parents. The decision to use ICT for social contact was therefore a choice made by parents, rather than a decision of the young adults themselves.

## 4 DISCUSSION

This systematic review suggests that the use of ICT for social contact by people with intellectual disabilities living in home care facilities may be feasible, even for populations for whom consumer technology is not usually usable. Our review, guided by four central research questions, identified nine studies on the use of virtual social contact by people with disabilities living in care facilities.

Three themes were described in this study: (a) means of ICT used for social contact; (b) effects on well‐being; and (c) benefits, barriers, and preconditions. Although our search was focused broadly on people with disabilities, all included studies targeted people with intellectual disabilities. Telephone and video were identified as direct reciprocal means of communication, and text messaging, social media, and email were identified as indirect means of communication. Regular means of communication were used across levels of disability. Use of the telephone and video have shown to be accessible and enjoyable even for single cases of persons with severe multiple disabilities if provided with intensive training and adaptive technology (Lancioni et al., 2015; Lancioni, Singh, O'Reilly, Sigafoos, Oliva, & Campodonico, 2013; Lancioni, Singh, O'Reilly, Sigafoos, Oliva, Campodonico, D'Amico, et al., 2013). The use of video conferencing by people with mild‐to‐moderate intellectual disabilities was briefly mentioned by one study (Ramsten et al., 2019), but no further details were reported. The modes of communication described in the included studies are also used by the general population (e.g., Van den Berg et al., 2012) and by long‐distance families who do not have regular in‐person contact (Abel et al., 2020; Wilding, 2006).

People with mild‐to‐moderate intellectual disabilities used consumer technology for social contact. This may have benefits, such as social inclusion, and may be easier for social care organisations to implement than specialised technology. Reported barriers were practical (e.g., difficulties using a device) or cognitive in nature (e.g., literacy problems, understanding jargon). Further, the included studies showed mixed evidence with regard to well‐being and quality of life, indicating potentially positive (e.g., positive moods, decreasing loneliness) and negative effects (e.g., frustration caused by practical difficulties). However, findings from the included studies were descriptive and there was considerable risk of bias in most of the studies (see Section 2 and the Appendix). Opportunities and barriers described in the studies have been mentioned by previous reviews on the use of ICT by people with intellectual disabilities (Borgström et al., 2019; Caton & Chapman, 2016; Chadwick et al., 2013). In the general population, the use of ICT for social contact with close others during the COVID‐19 pandemic has been associated with more positive affect, but also more negative affect and stress (Tibbetts et al., 2021). This may also be the case for people with intellectual disabilities. More empirical research is needed to make generalisable statements about the effects of using ICT for social contact on the well‐being of people with disabilities living in care facilities or supported living.

People with intellectual disabilities may need support with the use of ICT for social contact. This is important during the COVID‐19 pandemic, so that relationships with loved ones can be maintained (Tromans et al., 2020). However, most of the included studies indicated a lack of support by staff and family. People with intellectual disabilities have less access to ICT than the general population (‘digital divide’, Chadwick et al., 2013) and support is vital for learning how to use these technologies (e.g., Bryen et al., 2007; Carey et al., 2005; Li‐Tsang et al., 2005). Also, support may depend on organisational guidelines and the interests and attitudes from individual support staff and family members (Ramsten et al., 2019). Parsons et al. (2008) described that the implementation of ICT within service organisations for people with intellectual disabilities may not only depend on practical factors such as time, staff training, budget, and appropriate equipment, but also on the attitudes of support staff about the purpose, usefulness, and importance of ICT for the service users. For example, staff from organisations with little to no use of ICT did not consider the use of ICT appropriate for older users or those with more complex needs, whereas organisations with regular and enthusiastic use of ICT believed that it was a valuable tool that could flexibly be used with anyone (Parsons et al., 2008). Such beliefs, also from family members, are important to address when implementing ICT for social contact within care facilities.

Ramsten et al. (2019) emphasised the importance of support with the use of social media by people with intellectual disabilities because of exposure to risks such as victimisation and exploitation. Research of these risks is limited, but previous studies mentioned cyberbullying and financial and sexual exploitation (Buijs et al., 2016; Holmes & O'Loughlin, 2014). A recent study (Lee et al., 2020) showed that adolescents without intellectual disabilities who received fewer ‘likes’ on social media experienced stronger feelings of rejection and negative affect, especially those who were already victimised. People with intellectual disabilities may be vulnerable to these experiences. However, use of social media can be an important way of maintaining social relationships and forming new ones during times in which in‐person social activities are limited. A recent study (Sun et al., 2020) showed that active use of social media (e.g., commenting on messages or photos) during the COVID‐19 pandemic was positively associated with well‐being. Future studies should look into ways in which staff from care facilities can offer support for safe use of social media, for example through training or psychoeducation (Caton & Chapman, 2016).

### 4.1 Strengths and limitations

This study used a rigorous, two‐step approach to systematically review the evidence on the use and feasibility of ICT for social contact by people with disabilities living in care facilities. A potential limitation is that the researchers' perspective has guided the selection of literature and extraction of data and no stakeholders were involved in the interpretation of the findings. To improve objectivity in the interpretation of the findings, the data were extracted by two independent reviewers. In addition, the nine studies included in the review were conducted by only five research groups, which may have led to bias in the findings. Lastly, only articles in English, Dutch, and German were eligible for inclusion, therefore potentially relevant studies in other languages may have been missed.

### 4.2 Implications and directions for future studies

Previous studies have focused on the use of ICT to support daily life skills (e.g., Collins & Collet‐Klingenberg, 2017; Den Brok & Sterkenburg, 2015; Morash‐Macneil et al., 2017) but little attention has been paid to the use of ICT for social contact. None of the studies included in this review were conducted in the context of restricted in‐person visiting policies. Therefore, it is uncertain whether reported effects on well‐being can be generalised to situations in which digital contact is offered as a surrogate for in‐person contact. A recent study by Araten‐Bergman and Shpigelman (2021) involving 108 family caregivers of adults with developmental disabilities living in supported living showed that most families used digital technologies to stay in contact during the COVID‐19 pandemic. Family caregivers reported that the use of technology enabled them to provide emotional support, but that possibilities to provide significant social support were limited. These findings suggest that virtual social contact may not be regarded as a full alternative to in‐person contact, but that it may offer possibilities for social contact when in‐person contact is limited or restricted. The findings from the included studies (all conducted before the pandemic) may not fully apply to the context of a pandemic. During a pandemic, the use of ICT for social contact may be especially important for people living in care facilities, because of potential restrictions on social activities such as school and day activities. On the other hand, people with intellectual disabilities have a right to participate in activities that go on in society, and those activities increasingly migrate to the digital world. Because people with intellectual disabilities experience more barriers with the use of ICT compared to people without intellectual disabilities, they are at risk to be left out (Wottiez et al., 2018) and to be in double jeopardy when in‐person contact is restricted like in the current pandemic, when others may more easily switch to online communication. The use of ICT for social contact may not only be beneficial for people with intellectual disabilities, but also for their family, friends, and other social network members. This is an important area of research that needs to be examined and developed, also after the COVID‐19 pandemic. In addition, future work might explore the use of ICT for social contact by people with intellectual disabilities not living in home care facilities.

Based on a lack of attention to video conferencing in the included studies, it is still unknown whether its use is feasible for people with different types and levels of disabilities. Recently, Zaagsma et al. (2020) suggested that video conferencing is feasible and valuable for support for people with intellectual disabilities, which was increasingly used since the outbreak of the COVID‐19 pandemic. Some participants with mild intellectual disabilities in the study by Embregts et al. (2020) found video conferencing a positive way to maintain contact with friends and family during the pandemic, whereas others reported having negative feelings towards it, such as feeling more distant from others. Investigating the feasibility of video conferencing for the wider population of people with intellectual or multiple disabilities is an important direction for future studies.

Three studies included in this review suggested that even people with severe intellectual and multiple disabilities could enjoy using the telephone to call their loved ones and to watch videos of family members talking, even though their responses during these interactions were minimal. In addition, these technologies were perceived positively by family members (Lancioni et al., 2015; Lancioni, Singh, O'Reilly, Sigafoos, Oliva, & Campodonico, 2013; Lancioni, Singh, O'Reilly, Sigafoos, Oliva, Campodonico, D'Amico, et al., 2013). Recently, Dyzel et al. (2020) pointed at the need for more research on the usability of assistive technologies for people with deafblindness, including user experiences. Our findings emphasise that more research on the use of specialised technology for facilitating virtual social contact is needed for the broader population of people with intellectual and multiple disabilities. In addition, future studies might benefit from the participation of end users and experts of experience to identify or develop ways to address the needs, preferences, and implementation of virtual social contact by this population.

This review did not provide concrete insights about the requirements for effective implementation of ICT for social contact. The costs of equipment and an internet connection and a lack of technology support have been identified as impeding factors for the use and implementation of eHealth in support of people with intellectual disabilities (Frielink et al., 2020) and might also play a role here. In addition, support from staff and family appeared an important precondition for (independent) use of technology, but this was often lacking in the included studies. None of the included studies used parents or other family members as informants. Therefore, it is not known what family members might need to support their loved one with using virtual social contact. Future research should look into the preconditions for the implementation of ICT for social contact within care facilities, focusing both on practical conditions and support needs of people with intellectual disabilities, as well as the needs and beliefs of family members and support staff.

Lastly, all of the included studies focused on visual and auditory means of ICT‐mediated communication. However, the use of these technologies may not be feasible for some populations, such as people with visual and hearing impairments. In addition, regular communication technologies such as the telephone and video conferencing may not provide a ‘feeling of presence’, due to the absence of touch and the inability to make real eye contact (Cohen et al., 2017). Recent technological advances by Cohen et al. showed that 3D virtual reality and haptic technology can be used to recreate a feeling of presence, which might be expected to increase the positive emotional impact of such contact (Petrova & Schulz, 2021). Making such technologies available for the wider population would be a valuable direction for innovation and research.

## 5 CONCLUSION

This systematic review showed that the use of virtual social contact may be feasible and enjoyable for people with various types and levels of disabilities living in care facilities. However, there is very little research addressing the question whether this type of social contact meets the needs of people with disabilities and their families and friends, and how it might affect well‐being when in‐person contact is (temporarily) not possible, such as during quarantine situations or due to other circumstances (e.g., when elderly parents are unable to visit their children with disabilities). Given the impact that pandemic restrictions appear to have (e.g., Schuengel et al., 2020), more empirical evidence is needed about the feasibility and effectiveness of ICT for social contact of people with disabilities living in care facilities, before this might be recommended as an alternative for in‐person contact with loved ones, both during and after the COVID‐19 pandemic.

## Supporting information


**DATA S1:** Supporting Information

## Data Availability

Data sharing not applicable to this article as no datasets were generated or analysed during the current study.

## Quality assessment of included studies

The mixed methods appraisal tool (MMAT; Hong et al., 2018) was used to assess the risk of bias of the individual studies. The MMAT includes five domains: qualitative studies, randomised controlled trials, non‐randomised studies, quantitative descriptive studies, and mixed methods studies. Each domain has seven criteria: two screening criteria, assessing the clarity of research questions and whether the collected data allow to address the research questions, and five domain‐specific criteria. All studies included in this systematic review were observational studies.

The screening criteria were met by all but one study: the research questions in the study by Lancioni et al. (2015) were considered unclear, and therefore it was not possible to tell whether the collected data allowed to address the research questions. The studies by Patterson and Potter (2009), Ramsten et al. (2020), and Shpigelman (2018) met 100% of the quality criteria for qualitative studies. The study by Ramsten et al. (2019) lacked coherence between the data analysis and interpretation of the results because the quotes from support staff were not directly presented in the text but only used as examples for the identified themes. Therefore, this study met 80% of the criteria for qualitative studies.

The study by Shpigelman and Gill (2014) met 80% of the criteria for qualitative studies and 20% of the criteria for quantitative descriptive studies. The sampling strategy followed from the research question, but the sample was not representative of the target population. This limitation was discussed by the authors. The instruments for the quantitative part of the study were not clearly described. The survey (20 questions about Facebook use) was designed by the authors, but no reliability and validity data were reported, it was not clear how the survey questions were derived, and the four‐point scale of the multiple choice questions was not defined. The sample was a convenience sample. Thirteen participants were excluded from the study but no non‐response analysis was conducted. Further, the statistical analyses were not justified and it was not clear why non‐parametric tests were used.

The study by Parsons et al. (2006) met 20% of the criteria for qualitative studies. The qualitative approach (observations, interviews) was considered appropriate to answer the research questions. However, a quantitative descriptive approach might have been a more systematic method for getting an overview of different uses of ICT within care facilities. We were unable to tell whether the findings were adequately derived from the data (the paper describes ‘careful analysis of the data’ but no further details are provided) and whether the interpretation of the results was sufficiently substantiated by data (no quotes from interviews were provided, and it was unclear whether specific results were substantiated by observational or interview data). Further, some claims made in the discussion are speculative and do not seem to be linked to specific study findings. It should also be noted that the sample characteristics were not described.

Lastly, the studies by Lancioni, Singh, O'Reilly, Sigafoos, Oliva, Campodonico, D'Amico, et al. (2013); Lancioni, Singh, O'Reilly, Sigafoos, Oliva, and Campodonico (2013); Lancioni et al. (2015) met 20% of the criteria for quantitative descriptive studies. In all of these studies, the sampling strategy was not described and it was not clear whether the participants were representative of the target population. The participants in each case study appeared to meet the authors' own criteria (i.e., persons with multiple disabilities emerging from a minimally conscious state), but given the small samples, we were unsure as to whether the participants were representative of the target population. Also, we could not tell whether the measurements were appropriate – some variables were justified (e.g., the frequencies of phone calls), but some outcomes may not have been reliably measured, such as the number of smiles as indications of happiness. In addition, no statistical analyses were performed in these studies, the results were merely descriptive, and no justifications were provided for the statistical approaches. Therefore, we were unable to tell whether the statistical approaches were appropriate for answering the research questions.
