# Supplementary material for: People with intellectual disabilities living in care facilities engaging in virtual social contact: A systematic review of the feasibility and effects on well‐being
Source: J Appl Res Intellect Disabil. 2021 Jul 9;35(1):60–74. doi: 10.1111/jar.12926 (PMC9291764; doi:10.1111/jar.12926)
Supplement: Supplementary file 1 — DATA S1: Supporting Information [file JAR-35-60-s001.docx]

**Search protocol “People with intellectual disabilities living in care facilities engaging in virtual social contact: A systematic review of the feasibility and effects on well-being”**

**Formal screening**

Total before removing duplicates: *k* = 1038 articles

Total after removing duplicates: *k* = 891 articles

(5 June 2020)

**Scopus**

P, I, C, O: 202 articles (5 June 2020); 229 articles (15 January 2021; no additional eligible studies)

( TITLE-ABS-KEY ( ( "intellectual*" W/3 ( "disab*" OR "impair*" OR "handicap*" OR "subnormal*" OR "deficien*" ) ) OR ( "mental*" W/3 ( "disab*" OR "impair*" OR "handicap*" OR "subnormal*" OR "deficien*" OR "retard*" ) ) OR ( "learning" W/3 ( "disab*" OR "impair*" OR "difficult*" OR "deficien*" ) ) OR "moron*" OR "imbecile*" OR "feeble?minded" OR "subnormal*" OR "mental retard*" OR ( "Smith?Magenis" OR "Rett*" OR "Lesch?Nyhan" OR "Prader?Willi" OR "Angelman" OR "fragile?X" OR "Cri?du?chat" OR "Cornelia?de?Lange" OR "de?Lange" OR "Rubinstein?Taybi" OR "velocardiofacial" OR "DiGeorge" OR ( down* W/2 syndrome ) ) OR "developmental* delay*" OR "special?needs" OR "global developmental delay" OR "special educational needs" OR "social communication disorder" OR "chromosome 5p deletion" OR "5p minus" OR "Lejeune's" OR "cats cry" OR "5p syndrome" OR "crying cat" OR "17p- syndrome" OR "complex needs" OR "severe cerebral palsy" OR "multipl*-disab*" OR "cognitive-disabilit*" OR "profound?and?multiple" OR ( "cognitive-impairment*" AND NOT ( "dement*" OR "alzheimer*" OR "parkinson" OR "psychiatr*" OR "older" OR "aged" OR "elderly" ) ) OR "acquired?brain?injury" OR "traumatic?brain?injury" OR "deaf-blindness*" OR "deaf-blind" OR "deafblind" OR "deaf and blind" OR "Blind-Deaf Disorders" OR "Blindness-Deafness" OR "Deaf-Blind Syndromes" OR "Deaf-Blindness Disorders" OR "Deaf-Mutism-Blind Disorders" OR "Deafness-Blindness" OR "Hearing and Vision Loss" OR "Prelingual Deaf-Blind Disorders" OR "Prelingual Deafness-Blindness" OR "Vision and Hearing Loss" OR "vis* and hear* loss" OR "vis* and hear* impair*" OR "charge syndrome" OR "usher syndrome" OR "dual sensory loss" OR "multi-sensory impair*" ) ) AND ( TITLE-ABS-KEY ( "telecommunications" OR "electronic mail" OR "e-mail" OR "email" OR "radar" OR "radio" OR "satellite communications" OR "telefacsimile" OR "telephone" OR "phon*" OR "television" OR "videoconferencing" OR "video conferencing" OR "video?call*" OR "wireless technology" OR "assistive technology" OR "specialist communication software" OR "communication software" OR "communication technology" OR "augmentative and alternative communication" OR "text messaging" OR "whatsapp" OR "sms" OR "video call*" OR "facebook" OR "social media" OR "facetime" OR "messenger" OR "instagram" OR "snapchat" OR "wechat" OR "weibo" OR "tencent" OR "photo?voice" OR "app" OR "app's" OR "apps" ) ) AND ( TITLE-ABS-KEY ( "residential" OR "institution" OR "care facilit*" OR "care home" OR "nursing home" OR "nursing facilit*" OR "living arrangement" OR "lockdown" OR "quarantine*" OR "isolation" OR "isolat*" OR "confinement" OR "social distanc*" OR "physical distanc*" OR "shield*" OR "spacial distanc*" OR "infection" OR "infected" OR "infective" OR "infectious" OR "communicable" OR "COVID" OR "COVID-19" OR "nCoV" OR "coronavirus" OR "MERS" OR "SARS" OR "outbreak" OR "epidemic" OR "pandemic" OR "H1N1" OR "influenza" OR "norovirus" ) ) AND ( TITLE-ABS-KEY ( "quality of life" OR "mental health" OR "well-being" OR "psychological well-being" OR "emotional well-being" OR "happiness" OR "loneliness" OR "relationship quality" OR "interpersonal relation*" OR "social participation" OR "social relation*" OR "friendship*" OR "family" OR "Interpersonal Interactions" OR "enabling interpersonal relations" OR "Improving interpersonal relations" OR "social network" OR "social activities" OR "attachment NEAR relation*" OR "family NEAR relation*" OR "parent* NEAR relation*" OR "mother* NEAR relation*" OR "father* NEAR relation*" ) ) AND ( LIMIT-TO ( LANGUAGE , "English" ) OR LIMIT-TO ( LANGUAGE , "German" ) OR LIMIT-TO ( LANGUAGE , "Dutch" ) )

**Web of Science**

P, I, C, O: 67 articles (5 June 2020); 83 articles (15 January 2020; no additional eligible studies)

P = People with disability

TS=(( "intellectual*" NEAR/3 ( "disab*" OR "impair*" OR "handicap*" OR "subnormal*" OR "deficien*" ) ) OR ( "mental*" NEAR/3 ( "disab*" OR "impair*" OR "handicap*" OR "subnormal*" OR "deficien*" OR "retard*" ) ) OR ( "learning" NEAR/3 ( "disab*" OR "impair*" OR "difficult*" OR "deficien*" ) ) OR "moron*" OR "imbecile*" OR "feeble?minded" OR "subnormal*" OR "mental retard*" OR ( "Smith?Magenis" OR "Rett*" OR "Lesch?Nyhan" OR "Prader?Willi" OR "Angelman" OR "fragile?X" OR "Cri?du?chat" OR "Cornelia?de?Lange" OR "de?Lange" OR "Rubinstein?Taybi" OR "velocardiofacial" OR "DiGeorge" OR ( down* NEAR/2 syndrome ) ) OR "developmental* delay*" OR "special?needs" OR "global developmental delay" OR "special educational needs" OR "social communication disorder" OR "chromosome 5p deletion" OR "5p minus" OR "Lejeune's" OR "cats cry" OR "5p syndrome" OR "crying cat" OR "17p- syndrome" OR "complex needs" OR "severe cerebral palsy" OR "multipl*-disab*" OR "cognitive-disabilit*" OR "profound?and?multiple" OR ( "cognitive-impairment*" NOT ( "dement*" OR "alzheimer*" OR "parkinson" OR "psychiatr*" OR "older" OR "aged" OR "elderly" ) ) OR "acquired?brain?injury" OR "traumatic?brain?injury" OR "deaf-blindness*" OR "deaf-blind" OR "deafblind" OR "deaf and blind" OR "Blind-Deaf Disorders" OR "Blindness-Deafness" OR "Deaf-Blind Syndromes" OR "Deaf-Blindness Disorders" OR "Deaf-Mutism-Blind Disorders" OR "Deafness-Blindness" OR "Hearing and Vision Loss" OR "Prelingual Deaf-Blind Disorders" OR "Prelingual Deafness-Blindness" OR "Vision and Hearing Loss" OR "vis* and hear* loss" OR "vis* and hear* impair*" OR "charge syndrome" OR "usher syndrome" OR "dual sensory loss" OR "multi-sensory impair*")

I = ICT for social contact

TS=("telecommunications" OR "electronic mail" OR "e-mail" OR "email" OR "radar" OR "radio" OR "satellite communications" OR "telefacsimile" OR "telephone" OR "phon*" OR "television" OR "videoconferencing" OR "video conferencing" OR "video?call*" OR "wireless technology" OR "assistive technology" OR "specialist communication software" OR "communication software" OR "communication technology" OR "augmentative and alternative communication" OR "text messaging" OR "whatsapp" OR "sms" OR "video call*" OR "facebook" OR "social media" OR "facetime" OR "messenger" OR "instagram" OR "snapchat" OR "wechat" OR "weibo" OR "tencent" OR "photo?voice" OR "app" OR "app's" OR "apps")

C = In-person visits

TS=("residential" OR "institution" OR "care facilit*" OR "care home" OR "nursing home" OR "nursing facilit*" OR "living arrangement" OR "lockdown" OR "quarantine*" OR "isolation" OR "isolat*" OR "confinement" OR "social distanc*" OR "physical distanc*" OR "shield*" OR "spacial distanc*" OR "infection" OR "infected" OR "infective" OR "infectious" OR "communicable" OR "COVID" OR "COVID-19" OR "nCoV" OR "coronavirus" OR "MERS" OR "SARS" OR "outbreak" OR "epidemic" OR "pandemic" OR "H1N1" OR "influenza" OR "norovirus")

O = Quality of life

TS=("quality of life" OR "mental health" OR "well-being" OR "psychological well-being" OR "emotional well-being" OR "happiness" OR "loneliness" OR "relationship quality" OR "interpersonal relation*" OR "social participation" OR "social relation*" OR "friendship*" OR "family" OR "Interpersonal Interactions" OR "enabling interpersonal relations" OR "Improving interpersonal relations" OR "social network" OR "social activities" OR "attachment NEAR relation*" OR "family NEAR relation*" OR "parent* NEAR relation*" OR "mother* NEAR relation*" OR "father* NEAR relation*")

**PubMed (MEDLINE)**

P, I, C, O: 103 articles (5 June 2020); 153 articles (15 January 2021; no additional eligible studies)

(Intellectual Disability [MeSH] OR ( “intellectual”[Title/Abstract] AND (“disab*” [Title/Abstract] OR “impair*” [Title/Abstract] OR “handicap*” [Title/Abstract] OR “subnormal*” [Title/Abstract] OR “deficien*” [Title/Abstract] ) ) OR ( “mental*” [Title/Abstract] AND ( “disab*” [Title/Abstract] OR “impair*” [Title/Abstract] OR “handicap*”[Title/Abstract] OR “subnormal*” [Title/Abstract] OR “deficien*” [Title/Abstract] OR “retard*” [Title/Abstract] ) ) OR ( “learning” [Title/Abstract] AND ( “disab*” [Title/Abstract] OR “impair*” [Title/Abstract] OR “difficult*” [Title/Abstract] OR “deficien*” [Title/Abstract] ) )OR “moron*” [Title/Abstract] OR “imbecile*” [Title/Abstract] OR “feeble minded” [Title/Abstract] OR "subnormal*" [Title/Abstract] OR "mental retard*"[Title/Abstract] OR ( "Smith Magenis"[Title/Abstract] OR “Smith-Magenis” [Title/Abstract] OR "Rett*" [Title/Abstract] OR "Lesch Nyhan"[Title/Abstract] OR “Lesch-Nyhan”[Title/Abstract] OR "Prader Willi" [Title/Abstract] OR “Prader-Willi”[Title/Abstract] OR "Angelman" [Title/Abstract] OR "fragile X" [Title/Abstract] OR “fragile-X”[Title/Abstract] OR "Cri-du-chat"[Title/Abstract] OR “Cri du chat”[Title/Abstract] OR "Cornelia de Lange" [Title/Abstract] OR “Cornelia-de-Lange”[Title/Abstract] OR "de Lange" [Title/Abstract] OR “de-Lange”[Title/Abstract] OR "Rubinstein Taybi"[Title/Abstract] OR “Rubinstein-Taybi”[Title/Abstract] OR "velocardiofacial" [Title/Abstract] OR "DiGeorge" [Title/Abstract] OR ( down* [Title/Abstract] AND syndrome [Title/Abstract] ) ) OR "developmental* delay*" [Title/Abstract] OR "special needs" [Title/Abstract] OR “special-needs”[Title/Abstract] OR "global developmental delay" [Title/Abstract] OR "special educational needs" [Title/Abstract] OR "social communication disorder"[Title/Abstract] OR “chromosome 5p deletion”[Title/Abstract] OR “5p minus”[Title/Abstract] OR “Lejeune’s”[Title/Abstract] OR “cats cry”[Title/Abstract] OR “5p syndrome”[Title/Abstract] OR “crying cat”[Title/Abstract] OR “17p-syndrome”[Title/Abstract] OR “complex needs”[Title/Abstract] OR “severe cerebral palsy”[Title/Abstract] OR “multipl*-disab*”[Title/Abstract] OR “cognitive-disabilit*”[Title/Abstract] OR “profound and multiple”[Title/Abstract] OR “profound-and-multiple”[Title/Abstract] OR (“cognitive-impairment*”[Title/Abstract] NOT (“dement*”[Title/Abstract] OR “alzheimer*”[Title/Abstract] OR “parkinson”[Title/Abstract] OR “psychiatr*”[Title/Abstract] OR “older”[Title/Abstract] OR “aged”[Title/Abstract] OR “elderly”[Title/Abstract]) ) OR “acquired brain injury”[Title/Abstract] OR “acquired-brain-injury”[Title/Abstract] OR “traumatic brain injury”[Title/Abstract] OR “traumatic-brain-injury”[Title/Abstract] OR “deaf-blindness*”[Title/Abstract] OR "deaf-blind"[Title/Abstract] OR “deafblind”[Title/Abstract] OR “deaf and blind”[Title/Abstract] OR “Blind-Deaf Disorders”[Title/Abstract] OR “Blindness-Deafness”[Title/Abstract] OR “Deaf-Blind Syndromes”[Title/Abstract] OR “Deaf-Blindness Disorders”[Title/Abstract] OR “Deaf-Mutism-Blind Disorders”[Title/Abstract] OR “Deafness-Blindness”[Title/Abstract] OR “Hearing and Vision Loss”[Title/Abstract] OR “Prelingual Deaf-Blind Disorders”[Title/Abstract] OR “Prelingual Deafness-Blindness”[Title/Abstract] OR “Vision and Hearing Loss”[Title/Abstract] OR “vis* and hear* loss”[Title/Abstract] OR “vis* and hear* impair*”[Title/Abstract] OR “charge syndrome”[Title/Abstract] OR “usher syndrome”[Title/Abstract] OR “dual sensory loss”[Title/Abstract] OR “multi-sensory impair*”[Title/Abstract]) AND (telecommunications [MeSH] OR “telecommunications”[Title/Abstract] OR "electronic mail"[Title/Abstract] OR “e-mail”[Title/Abstract] OR “email”[Title/Abstract] OR "radar"[Title/Abstract] OR "radio"[Title/Abstract] OR "satellite communications"[Title/Abstract] OR "telefacsimile"[Title/Abstract] OR "telephone"[Title/Abstract] OR “phon*”[Title/Abstract] OR "television"[Title/Abstract] OR "videoconferencing"[Title/Abstract] OR “video conferencing”[Title/Abstract] OR “video-call*”[Title/Abstract] OR “video call”[Title/Abstract] OR "wireless technology"[Title/Abstract] OR "assistive technology"[Title/Abstract] OR "specialist communication software"[Title/Abstract] OR "communication software"[Title/Abstract] OR "communication technology"[Title/Abstract] OR "augmentative and alternative communication"[Title/Abstract] OR "text messaging"[Title/Abstract] OR "whatsapp"[Title/Abstract] OR "sms"[Title/Abstract] OR "video call*"[Title/Abstract] OR “facebook”[Title/Abstract] OR “social media”[Title/Abstract] OR “facetime”[Title/Abstract] OR “messenger”[Title/Abstract] OR “instagram”[Title/Abstract] OR “snapchat”[Title/Abstract] OR “wechat”[Title/Abstract] OR “weibo”[Title/Abstract] OR “tencent”[Title/Abstract] OR “photo voice”[Title/Abstract] OR “photo-voice”[Title/Abstract] OR “app”[Title/Abstract] OR “app’s”[Title/Abstract] OR “apps”[Title/Abstract]) AND ("residential"[Title/Abstract] OR "institution"[Title/Abstract] OR "care facilit*"[Title/Abstract] OR "care home"[Title/Abstract] OR "nursing home"[Title/Abstract] OR "nursing facilit*"[Title/Abstract] OR "living arrangement"[Title/Abstract] OR "lockdown"[Title/Abstract] OR "quarantine*"[Title/Abstract] OR "isolation"[Title/Abstract] OR "isolat*"[Title/Abstract] OR "confinement"[Title/Abstract] OR "social distanc*"[Title/Abstract] OR "physical distanc*"[Title/Abstract] OR "shield*"[Title/Abstract] OR "spacial distanc*"[Title/Abstract] OR "infection"[Title/Abstract] OR "infected"[Title/Abstract] OR "infective"[Title/Abstract] OR "infectious"[Title/Abstract] OR "communicable"[Title/Abstract] OR "COVID"[Title/Abstract] OR "COVID-19"[Title/Abstract] OR "nCoV"[Title/Abstract] OR "coronavirus"[Title/Abstract] OR "MERS"[Title/Abstract] OR "SARS"[Title/Abstract] OR "outbreak"[Title/Abstract] OR "epidemic"[Title/Abstract] OR "pandemic"[Title/Abstract] OR "H1N1"[Title/Abstract] OR "influenza"[Title/Abstract] OR "norovirus"[Title/Abstract]) AND (quality of life [MeSH] OR mental health [MeSH] OR "quality of life"[Title/Abstract] OR "mental health"[Title/Abstract] OR "well-being"[Title/Abstract] OR "psychological well-being"[Title/Abstract] OR "emotional well-being"[Title/Abstract] OR "happiness"[Title/Abstract] OR "loneliness"[Title/Abstract] OR "relationship quality"[Title/Abstract] OR "interpersonal relation*"[Title/Abstract] OR “social participation”[Title/Abstract] OR “social relation*”[Title/Abstract] OR “friendship*”[Title/Abstract] OR “family”[Title/Abstract] OR “Interpersonal Interactions”[Title/Abstract] OR “enabling interpersonal relations”[Title/Abstract] OR “Improving interpersonal relations”[Title/Abstract] OR “social network”[Title/Abstract] OR “social activities”[Title/Abstract] OR (“attachment”[Title/Abstract] AND “relation*”[Title/Abstract]) OR (“family”[Title/Abstract] AND “relation*”[Title/Abstract]) OR (“parent*”[Title/Abstract] AND “relation*”[Title/Abstract]) OR (“mother*”[Title/Abstract] AND “relation*”[Title/Abstract]) OR (“father*”[Title/Abstract] AND “relation*”[Title/Abstract]))

**LENS.org**

P, I, C, O: 623 articles (5 June 2020); 713 articles (15 January 2020; no additional eligible studies)

abstract:(((intellectual AND (disab* OR impair* OR handicap* OR subnormal* OR deficien*)) OR (mental* AND (disab* OR impair* OR handicap* OR subnormal* OR deficien* OR retard*)) OR (learning AND (disab* OR impair* OR difficult* OR deficien*)) OR moron* OR imbecile* OR feeble?minded OR subnormal* OR (Smith?Magenis OR Rett* OR Lesch?Nyhan OR Prader?Willi OR Angelman OR fragile?X OR Cri?du?chat OR Cornelia?de?Lange OR de?Lange OR Rubinstein?Taybi OR velocardiofacial OR DiGeorge OR (down* AND syndrome)) OR "developmental* delay*" OR special?needs OR "global developmental delay" OR "special educational needs" OR "social communication disorder" OR "chromosome 5p deletion" OR "5p minus" OR Lejeune’s OR cats?cry OR "5p syndrome" OR crying?cat OR 17p-syndrome OR "complex needs" OR "severe cerebral palsy" OR multipl*-disab* OR cognitive-disabilit* OR "profound and multiple" OR profound-and-multiple OR (cognitive-impairment* NOT (dement* OR alzheimer* OR parkinson OR psychiatr* OR older OR aged OR elderly)) OR acquired?brain?injury OR traumatic?brain?injury OR deaf-blindness* OR deaf-blind OR deafblind OR "deaf and blind" OR "Blind-Deaf Disorders" OR Blindness-Deafness OR "Deaf-Blind Syndromes" OR "Deaf-Blindness Disorders" OR "Deaf-Mutism-Blind Disorders" OR Deafness-Blindness OR "Hearing and Vision Loss" OR "Prelingual Deaf-Blind Disorders" OR "Prelingual Deafness-Blindness" OR "Vision and Hearing Loss" OR "vis* and hear* loss" OR "vis* and hear* impair*" OR "charge syndrome" OR "usher syndrome" OR "dual sensory loss" OR "multi-sensory impair*") AND (telecommunications OR "electronic mail" OR e?mail OR radar OR radio OR "satellite communications" OR telefacsimile OR telephone OR phon* OR television OR videoconferencing OR video?conferencing OR video?call* OR "wireless technology" OR "assistive technology" OR "specialist communication software" OR "communication software" OR "communication technology" OR "augmentative and alternative communication" OR "text messaging" OR whatsapp OR sms OR facebook OR "social media" OR facetime OR messenger OR instagram OR snapchat OR wechat OR weibo OR tencent OR "photo?voice" OR app OR app’s OR apps) AND (residential OR institution OR "care facilit*" OR "care home" OR "nursing home" OR "nursing facilit*" OR "living Arrangement" OR lockdown OR quarantine* OR isolation OR isolat* OR confinement OR "social distanc*" OR "physical distanc*" OR shield* OR "spacial distanc*" OR infection OR infected OR infective OR infectious OR communicable OR COVID OR COVID-19 OR nCoV OR coronavirus OR MERS OR SARS OR outbreak OR epidemic OR pandemic OR H1N1 OR influenza OR norovirus) AND ("quality of life" OR "mental health" OR well-being OR "psychological well-being" OR "emotional well-being" OR happiness OR loneliness OR "relationship quality" OR "interpersonal relation*" OR "social participation" OR "social relation*" OR friendship* OR family OR "interpersonal interactions" OR "enabling interpersonal relations" OR "improving interpersonal relations" OR "social network" OR "social activities" OR (attachment AND relation*) OR (family AND relation*) OR (parent* AND relation*) OR (mother* AND relation*) OR (father* AND relation*)))

**IEEE Xplore**

P and I: 43 articles (5 June 2020); 47 articles (15 January 2021; no additional eligible studies)

*“Unable to find results” if we include C*

( ( "Abstract":"intellectual*" NEAR ( "Abstract":"disability" OR "Abstract":"disabilities" OR "Abstract":"disabled" OR "Abstract":"impairment" OR "Abstract":"impairments" OR "Abstract":"impaired" OR "Abstract":"handicap" OR "Abstract":"handicaps" OR "Abstract":"handicapped" OR "Abstract":"deficiency" OR "Abstract":"deficiencies" OR "Abstract":"deficient") ) OR ( "Abstract":"mental*" NEAR ( "Abstract":"disability" OR "Abstract":"disabilities" OR "Abstract":"disabled" OR "Abstract":"impairment" OR "Abstract":"impairments" OR "Abstract":"impaired" OR "Abstract":"handicap" OR "Abstract":"handicaps" OR "Abstract":"handicapped" OR "Abstract":"deficiency" OR "Abstract":"deficiencies" OR "Abstract":"deficient" OR "Abstract":"retardation" OR "Abstract":"retard" ) ) OR ( "Abstract":"learning" NEAR ( "Abstract":"disability" OR "Abstract":"disabilities" OR "Abstract":"impairment" OR "Abstract":"impairments"OR "Abstract":"handicap" OR "Abstract":"handicaps" OR "Abstract":"deficiency" OR "Abstract":"deficiencies" OR "Abstract":"deficient") ) OR "Abstract":"down syndrome" OR "Abstract":"multiple disab*" OR "Abstract":"cognitive disab*" OR "Abstract":"acquired brain injury" OR "Abstract":"traumatic brain injury" OR "Abstract":"deaf-blindness" OR "Abstract":"deaf-blind" OR "Abstract":"deafblind" OR "Abstract":"deaf and blind" OR "Abstract":"Hearing and Vision Loss" OR "Abstract":"vision and hearing loss" OR "Abstract":"visual and hearing impairment" “Abstract":“dual sensory loss" OR "Abstract":"multi-sensory impairment" ) AND ( "Abstract":"telecommunications" OR "Abstract":"electronic mail" OR "Abstract":"e-mail" OR "Abstract":"email" OR "Abstract":"radar" OR "Abstract":"radio" OR "Abstract":"satellite communications" OR "Abstract":"telefacsimile" OR "Abstract":"telephone" OR "Abstract":"phone" OR "Abstract":"television" OR "Abstract":"videoconferencing" OR "Abstract":"video conferencing" OR "Abstract":"video?call*" OR "Abstract":"wireless technology" OR "Abstract":"assistive technology" OR "Abstract":"specialist communication software" OR "Abstract":"communication software" OR "Abstract":"communication technology" OR "Abstract":"augmentative and alternative communication" OR "Abstract":"text messaging" OR "Abstract":"whatsapp" OR "Abstract":"sms" OR "Abstract":"facebook" OR "Abstract":"social media" OR "Abstract":"facetime" OR "Abstract":"messenger" OR "Abstract":"instagram" OR "Abstract":"snapchat" OR "Abstract":"wechat" OR "Abstract":"weibo" OR "Abstract":"tencent" OR "Abstract":"app" OR "Abstract":"apps" )

C = In-person visits

AND ("Abstract":"residential" OR "Abstract":"institution" OR "Abstract":"care facility" OR "Abstract":“care facilities” OR "Abstract":"care home" OR "Abstract":"nursing home" OR "Abstract":"nursing facility" OR "Abstract":“nursing facilities” OR "Abstract":"living arrangement" OR "Abstract":"lockdown" OR "Abstract":"quarantine" OR "Abstract":"isolation" OR "Abstract":"isolated" OR "Abstract":"confinement" OR "Abstract":"social distance" OR "Abstract":“social distancing” OR "Abstract":"physical distance" OR "Abstract":“physical distancing” OR "Abstract":"shielding" OR "Abstract":"spatial distance" OR "Abstract":“spatial distancing” OR "Abstract":"infection" OR "Abstract":"infected" OR "Abstract":"infective" OR "Abstract":"infectious" OR "Abstract":"communicable" OR "Abstract":"COVID" OR "Abstract":"COVID-19" OR "Abstract":"nCoV" OR "Abstract":"coronavirus" OR "Abstract":"MERS" OR "Abstract":"SARS" OR "Abstract":"outbreak" OR "Abstract":"epidemic" OR "Abstract":"pandemic" OR "Abstract":"H1N1" OR "Abstract":"influenza" OR "Abstract":"norovirus")
